# Supplementary material for: Uncharacterized Proteins CxORFx: Subinteractome Analysis and Prognostic Significance in Cancers
Source: Int J Mol Sci. 2023 Jun 15;24(12):10190. doi: 10.3390/ijms241210190 (PMC10298883; doi:10.3390/ijms241210190)
Supplement: Supplementary file 1 [file ijms-24-10190-s001.zip › ijms-2404219-supplementary.pdf]

Supplementary material

Uncharacterized proteins CxORFx: subinteractome analysis and prognostic significance in cancers

Pavel Ershov, Evgeniy Yablokov, Yuri Mezentsev, and Alexis Ivanov
Institute of Biomedical Chemistry, Moscow, Russian Federation

Correspondence to: pavel79@inbox.ru (Pavel Ershov)

Table S1. Map of prognostic significance of ORF-gene expression patterns in different cancer types*

**Genes**	**breast cancer**	**cervical cancer**	**colorectal cancer**	**endometrial cancer**	**glioma**	**head and neck cancer**	**liver cancer**	**lung cancer**	**melanoma**	**ovarian cancer**	**pancreatic cancer**	**prostate cancer**	**kidney cancer**	**testicular cancer**	**thyroid cancer**	**urothelial carcinoma**
C10orf88							●									
C11orf1				●									●			
C11orf24					●			●					●			
C11orf49							●									
C11orf71				●									●			
C11orf96													●			
C12orf49							●						●			
C12orf73							●									●
C14orf119							●								●	
C14orf132											●					●
C15orf39									●				●	●		
C15orf40													●			
C15orf61													●			
C15orf65				●												
C16orf71				●												
C16orf74						●			●		●					
C16orf86											●		●			
C16orf87						●										
C17orf47													●			
C17orf58							●						●			
C18orf21							●			●						
C19orf18				●												
C19orf47	●			●			●									
C19orf57				●		●										
C19orf71						●					●		●			
C1orf109							●									
C1orf112				●												
C1orf115				●						●						
C1orf123													●			
C1orf131													●			●
C1orf159																
C1orf162													●			
C1orf174							●		●				●			
C1orf194				●												
C1orf198													●			

C1orf21													●			
C1orf210													●			
C1orf216							●						●			
C1orf226				●									●			
C1orf50									●							
C1orf52							●					●	●			
C1orf53				●												
C1orf54													●			
C20orf194											●		●			
C20orf27							●						●			
C20orf96							●				●					●
C21orf58						●			●		●					
C21orf62													●			
C22orf15				●												
C22orf39				●									●			
C2orf42											●					
C2orf54				●												
C2orf70											●					
C2orf72											●		●			
C2orf76													●			
C2orf88				●					●				●			
C3orf38										●		●				
C3orf62	●					●	●	●			●					●
C3orf70													●			
C4orf19													●			
C4orf36													●			
C4orf47													●			
C5orf24													●			
C5orf46		●											●			
C5orf51							●									
C6orf120		●					●			●						
C6orf132											●					
C6orf136													●			●
C6orf141	●			●												
C6orf203											●		●			●
C6orf226	●										●		●			
C7orf31					●											
C8orf33	●						●									
C8orf59													●			
C8orf76	●			●									●			
C8orf82													●			
C8orf88									●							●
C9orf116				●												
C9orf152				●												
C9orf16		●	●				●									

C9orf40							●									
C9orf64													●			
CXorf40B															●	
**Total**	**6**	**3**	**1**	**20**	**2**	**6**	**20**	**2**	**7**	**4**	**14**	**2**	**43**	**1**	**2**	**8**

*according to data from The Human Proteome Atlas portal (https://www.proteinatlas.org/)

Table S2. Correlations between the gene expression patterns and tumor immune cell infiltration*

Cancer type	Gene name	Immune cell type	Correlation**
THYM	*C10orf25*	DC	0.73
	C12orf42	$CD4^{+}$, DC	0.77
	C16orf54	BC, $CD4^{+}$, DC	0.72 - 0.75
	C17orf67	$CD4^{+}$	0.81
	C17orf67	DC	0.82
	C21orf58	BC, $CD4^{+}$, DC	0.72 - 0.78
	C4orf46	BC	0.72
	C9orf40	BC, DC	0.72 - 0.73
	CXorf65	$CD4^{+}$	0.79
LGG	*C16orf54*	$CD4^{+}$	0.82
	C16orf54	DC, M, N	0.73 - 0.84
	C1orf162	$CD4^{+}$, DC, M, N	0.7 - 0.78
	CXorf21	$CD4^{+}$, DC, N	0.73 - 0.77
SKCM, mSKCM	*C16orf54*	DC	0.74
	C1orf162	DC	0.75 - 0.76
	CXorf21	DC	0.73
	CXorf21	N	0.78 - 0.81
SARC	*C16orf54*	DC	0.72
	C1orf162	M	0.74 - 0.76
BRCA	*C16orf54*	DC	0.73
	C1orf162	DC	0.73
HNSC	*C11orf21*	$CD8^{+}$	0.77
	CXorf21	N	0.74
LUAD	*C1orf162*	DC	0.74

*Only those types of cancer in which an additional correlation was revealed between the infiltration of cancer immune cells and cumulative survival are shown (log-rank test p-value < 0.05).
**log-rank test p-value < 0.001, cut-off value of purity-corrected partial Spearman's rho value > 0.7.
Abbreviations: BC, B cells; $CD4^{+}$, $CD4^{+}$ T-cells; $CD8^{+}$, $CD8^{+}$ T-cells; DC, dendritic cells; M, macrophages; N, neutrophils; THYM - thymic carcinoma; LGG – low grade glioma; SKCM – primary or metastatic (m) skin cutaneous melanoma; SARC – sarcoma; BRCA – breast cancer; HNSC - head and neck cancer; LUAD – lung adenocarcinoma.

Table S3. Contextual analysis of subinteractomes of ORF-proteins

ORF-protein	Differential gene expression pattern in cancers	A set of protein partners associated with cancer hallmarks (CH)			
		CH-associated protein partners	KEGG pathways enrichment analysis* (≥ 2 terms)	Co-expression tissue-specific analysis (Spearmen correlation coefficient**, cut-off value = 0.5)	PPI clusters within CH-associated protein partners***
C1orf21	LAML ↑[#]<br>PAAD ↑[#]<br>THYM ↑<br>UCEC ↓	CSNK1G1, **ESR1**, **HRAS**, MAGEA3, MOV10, PPP2CA, **PPP2R1A**, PPP2R1B, PPP2R5A, PPP2R5B, SHMT2	Pathways in cancer<br>Breast cancer<br>Pancreatic cancer<br>PD-L1 expression and PD-1 checkpoint pathway in cancer<br>Colorectal cancer<br>Proteoglycans in cancer<br>Acute myeloid leukemia<br>Endometrial cancer	PPP2R5A (0.53) pancreatic cancer	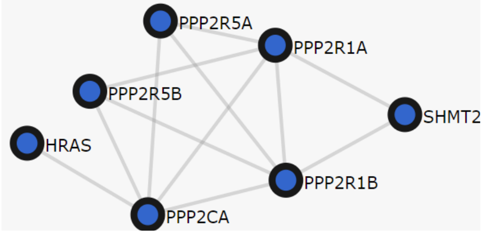
C1orf115	TGCT ↓	CTR9, **FBXO11**, FBXO7, **MDM2**, NCS1, NRBF2, PRMT5, TOP2A, **UBR5**	Hepatocellular carcinoma<br>Transcriptional misregulation in cancer<br>Human T-cell leukemia virus 1 infection	MDM2 (0.51) testis<br>NCS1 (0.62) testis<br>NRBF2 (0.55) testis	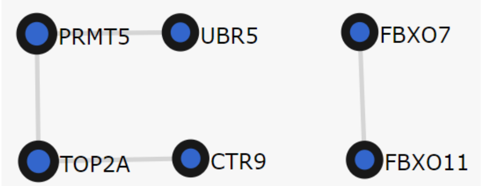
C1orf123	DLBC ↑<br>THYM ↑	AGRN, ANXA1, C1QBP, CALU, **CDKN1A**, **CLTC**, DAB2, **HEY1**, **RAF1**, UBA6**,** USP5	Proteoglycans in cancer<br>Pathways in cancer<br>Non-small cell lung cancer<br>Colorectal cancer<br>Pancreatic cancer	AGRN (0.50) thymoma<br>HEY1 (0.64) thymoma	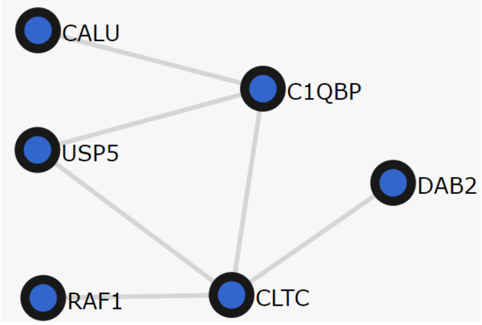

C1orf131	DLBC ↑<br>THYM ↑	AIMP2, APP, **CDC73,** DDX21, EIF3L, EPRS1, GEM, IARS1, ID2, IQGAP1, **JUN,** KARS1, **KRAS,** MYBBP1A, NUMB, **PPP2R1A,** QARS1, RARS1, RPA1, **SMARCB1,** SMARCC1	Gastric cancer<br>Breast cancer<br>Pathways in cancer<br>Colorectal cancer<br>Chronic myeloid leukemia<br>Prostate cancer	APP (0.65) Diffuse large B cell lymphoma (DLBC)<br>CDC73 (0.73) thymoma<br>CDC73 (0.77) DLBC<br>DDX21 (0.81) DLBC<br>DDX21 (0.7) thymoma<br>GEM (0.69) DLBC<br>IARS1 (0.67) thymoma<br>IARS1 (0.81) DLBC<br>ID2 (0.59) DLBC<br>IQGAP1 (0.67) thymoma<br>IQGAP1 (0.76) DLBC<br>JUN (0.61) DLBC<br>KRAS (0.77) DLBC<br>KRAS (0.75) thymoma<br>MYBBP1A (0.57) DLBC<br>NUMB (0.79) DLBC<br>RPA1 (0.61) thymoma<br>SMARCC1 (0.65) DLBC<br>SMARCC1 (0.66) thymoma	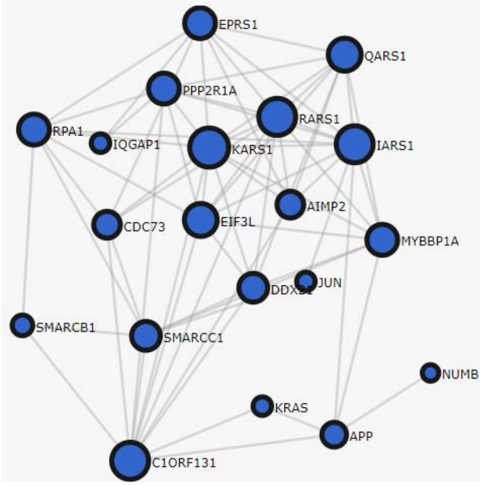
C1orf198	THYM ↑	CDK2, APEX1, ARHGEF1, BID, **CDH1,** CTSB, DAAM1, ELAVL1, ENO1, **EZR,** HSPA1A, HSPA8, KLF13, NCSTN, PDP1, PLEKHA7, **RHOA,** **XPO1**	Proteoglycans in cancer<br>Pathways in cancer<br>Endometrial cancer<br>Gastric cancer<br>Pancreatic cancer<br>Non-small cell lung cancer<br>Central carbon metabolism in cancer<br>Choline metabolism in cancer	CDH1 (0.65) thymoma<br>CTSB (0.54) thymoma<br>DAAM1 (0.65) thymoma<br>HSPA1A (0.67) thymoma<br>NCSTN (0.83) thymoma<br>PDP1 (0.64) thymoma<br>PLEKHA7 (0.69) thymoma<br>XPO1 (0.56) thymoma	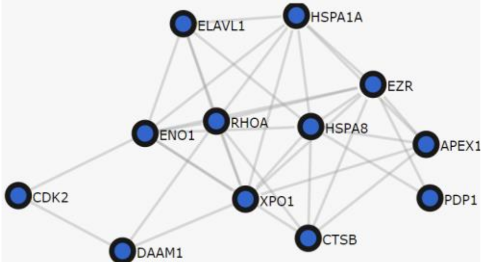

C1orf210	COAD ↑<br>OV ↑<br>READ ↑<br>SKCM ↓<br>UCES ↑<br>UCS ↑	ACTB, BTRC, CUL1, FBXW11, HMOX2, NR2F1, PTPN1, SKP1, SLC27A1, SLC27A4, SPAG9	Pathways in cancer<br>Prostate cancer<br>Prostate cancer<br>Chronic myeloid leukemia<br>Small cell lung cancer<br>Pancreatic cancer	SLC27A4 (0.71) colon	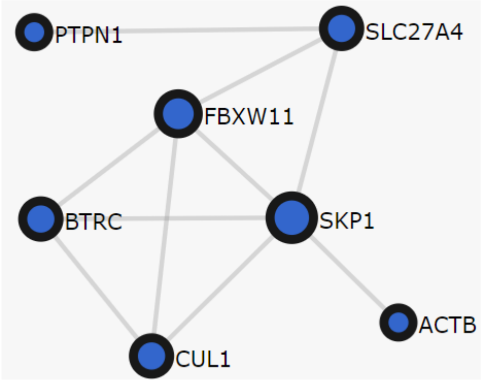
C1orf226	GBM ↑<br>LGG ↑	**BARD1, BCR, BRD3,** BTRC, BUB3, DYRK1A, ESR2, **FOXO3,** FYN, **HRAS,** NOS1, PLK1, PLK2, **PRCC,** PTPRJ, YWHAG	Pathways in cancer<br>Gastric cancer<br>MicroRNAs in cancer<br>Prostate cancer<br>Breast cancer<br>Viral carcinogenesis	BARD1 (0.55) low-grade glioma<br>BARD1 (0.51 – 0.54) brain<br>BCR (0.53 – 0.59) brain<br>BRD3 (0.52 – 0.75) brain<br>BTRC (0.6 – 0.76) brain<br>BUB3 (0.56 – 0.75) brain<br>DYRK1A (0.62 – 0.72) brain<br>ESR2 (0.55) brain<br>FOXO3 (0.55 – 0.57) brain<br>FYN (0.63 – 0.73) brain<br>PLK1 (0.57 – 0.78) brain<br>PRCC (0.54 - 0.79) brain<br>PTPRJ (0.53 - 0.79) brain<br>YWHAG (0.59 - 0.65) brain	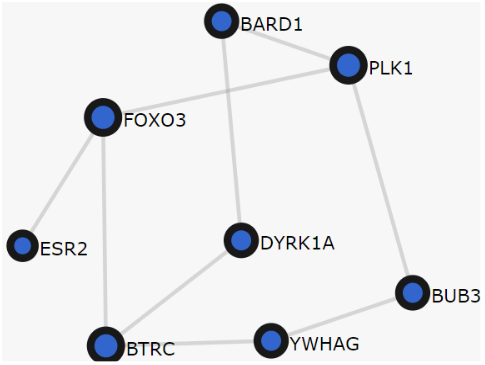
C2orf16	TGCT ↓	HNRNPU, ITCH, MAML3, **MYC**, PRC1, SPAG5, **THRAP3**	Transcriptional misregulation in cancer<br>Pathways in cancer<br>Small cell lung cancer<br>Kaposi sarcoma-associated herpesvirus infection<br>Breast cancer	ITCH (0.64) testis<br>SPAG5 (0.68) testis	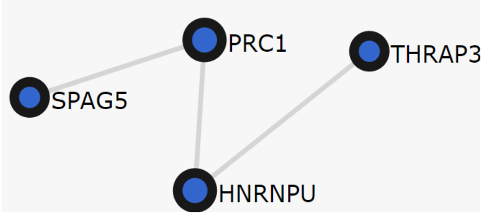

C2orf74	COAD ↓<br>DLBC ↑<br>LAML ↑<br>READ ↓<br>THYM ↑<br>UCES ↓	**POT1, PPP1CA, PPP1CC,** PPP1R3B, PPP1R9A, PPP1R9B, SCRIB, SHOC2, ZFYVE9	Proteoglycans in cancer<br>Pathways in cancer<br>Gastric cancer<br>Breast cancer<br>Endometrial cancer<br>Hepatocellular carcinoma	PPP1R9A (0.65) colon<br>PPP1R9A (0.57) thymoma<br>ZFYVE9 (0.64) thymoma	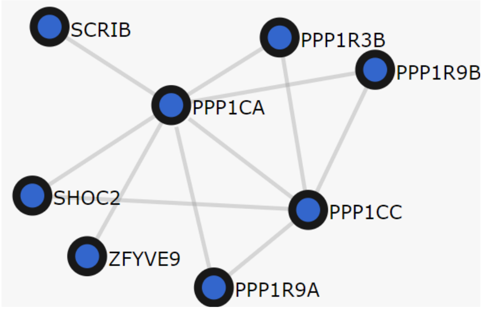
C2orf88	LAML ↓<br>LGG ↑<br>OV ↑<br>TGCT ↓	FBXO7, FBXW8, GPR161, **MYOD1, PRKACA,** PRKACB, **PRKAR1A,** PRKAR1B, PRKAR2B, TRIM25	Pathways in cancer<br>Proteoglycans in cancer<br>Glioma<br>Choline metabolism in cancer<br>Hepatocellular carcinoma<br>Prostate cancer<br>Colorectal cancer	FBXO7 (0.72) testis<br>FBXO7 (0.63 – 0.86) brain<br>FBXW8 (0.54) testicular germ cell cancer (TGCT)<br>FBXW8 (0.56 – 0.77) brain<br>GPR161 (0.58) TGCT<br>GPR161 (0.56) brain<br>PRKACA (0.53) TGCT<br>PRKACB (0.55) acute myeloid leukemia (LAML)<br>PRKACB (0.61 – 0.79) brain<br>PRKAR1A (0.62) testis<br>PRKAR1A (0.61 – 0.85) brain<br>PRKAR2B (0.64) brain<br>TRIM25 (0.61) brain	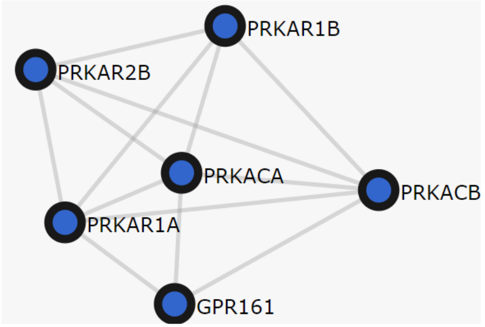
C3orf62	TGCT ↓	APP, BLK, CCN2, CREBBP, EP300, IDE	Pathways in cancer<br>Transcriptional misregulation in cancer<br>Prostate cancer<br>Gastric cancer<br>Pancreatic cancer<br>Breast cancer	CREBBP (0.59) testis<br>IDE (0.69) testis	Not found

C4orf17	TGCT ↓	CCN2, CHD3, COPS6, CRK, GRN, KAT2B, KDM1A, PRMT1	Transcriptional misregulation in cancer<br>Breast cancer<br>Chronic myeloid leukemia<br>Hepatocellular carcinoma<br>Prostate cancer	Not found	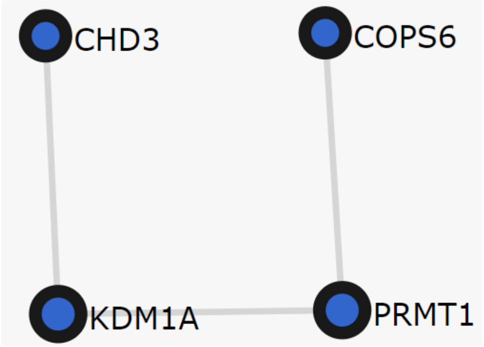
C4orf19	LAML ↑<br>READ ↑	**EIF3E,** NFKB1, PHF1, STK24,STK26**,** TRIM25	Pathways in cancer<br>Kaposi sarcoma-associated herpesvirus infection<br>Pancreatic cancer<br>Breast cancer	Not found	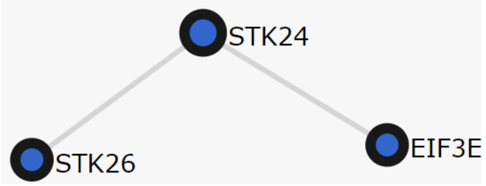
C4orf46	THYM ↑	APP, BLOC1S6, CDR2, DYNLL1, ELAVL1, HIF1AN, PICK1, RASSF1, SAV1, STK4	Pathways in cancer<br>Kaposi sarcoma-associated herpesvirus infection<br>Proteoglycans in cancer<br>Colorectal cancer<br>Viral carcinogenesis	BLOC1S6 (0.57) thymoma<br>CDR2 (0.6) hymoma<br>ELAVL1 (0.81) hymoma<br>STK4 (0.65) hymoma	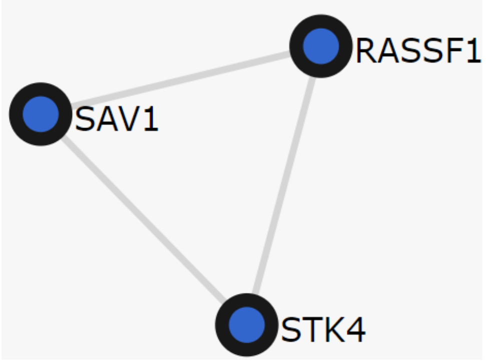

C5orf24	THYM ↑	PKNOX1, ATP2A1, WDR45B, BCAR1, MAPK9, FOXC1, MEIS1, AIM2, ARHGEF19, **XPO1**. **PBX1**, **MYC,** PYHIN1, **CIC, GOPC,** STRADA, CHFR, **CREB1, STK11**	Hepatocellular carcinoma<br>Pancreatic cancer<br>Transcriptional misregulation in cancer<br>Colorectal cancer<br>Gastric cancer<br>Breast cancer<br>Chronic myeloid leukemia	PKNOX1 (0.56) thymoma<br>WDR45B (0.85) thymoma<br>BCAR1 (0.6) thymoma<br>MAPK9 (0.79) thymoma<br>FOXC1 (0.73) thymoma<br>MEIS1 (0.68) thymoma<br>PBX1 (0.7) thymoma<br>CIC (0.72) thymoma<br>GOPC (0.85) thymoma<br>STRADA (0.76) thymoma<br>CREB1 (0.80) thymoma<br>STK11 (0.56) **thymoma**	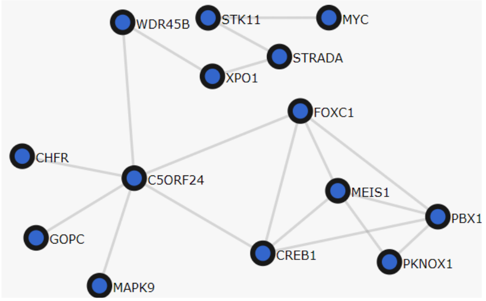
C6orf132	COAD ↑<br>KICH ↓<br>OV ↑<br>READ ↑<br>SKCM ↓<br>TGCT ↑<br>UCES ↑<br>UCS ↑	BAIAP2, **CDH1**, **ESR1**, **EZR**	Proteoglycans in cancer<br>Pathways in cancer<br>Breast cancer<br>Choline metabolism in cancer<br>MicroRNAs in cancer<br>Gastric cancer	BAIAP2 (0.62) kidney<br>CDH1 (0.58) TGCT<br>CDH1 (0.76) uterine carcinosarcoma (UCS)<br>CDH1 (0.6) colon sigmoid<br>CDH1 (0.92) colon transverse<br>CDH1 (0.82) kidney<br>ESR1 (0.66) kidney<br>EZR (0.71) TGCT<br>EZR (0.58) UCS<br>EZR (0.89) colon	Not found
C6orf222	READ ↑	WDR83, CDC16, CAPRIN1, MAPK9, ANAPC4, BCL2L2 PDLIM5, MAPK8, ANAPC7, YWHAG, CDC23, **BAX**, YWHAQ, USP7, GRPEL1, BCL2L1, PRKAA2, MAPK14, RBBP7, TCOF1,	MicroRNAs in cancer<br>Pancreatic cancer<br>Chronic myeloid leukemia<br>Pathways in cancer<br>Non-small cell lung cancer<br>Melanoma	MAPK14 (0.53) rectal adenocarcinoma (READ)	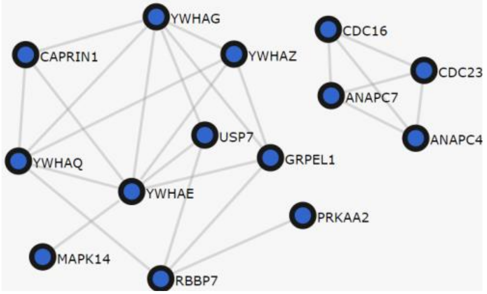

		MTHFR, YWHAZ, **YWHAE**, BNIP2			
C8orf48	TGCT ↓	**CCNE1**, **FGFR3**, GRIN2C, **HRAS**, KDM1A, MCC, MDFI, OPTN, PRPS1, RNF11, TARDBP, **TSC1**	Gastric cancer<br>Breast cancer<br>Hepatocellular carcinoma<br>Pancreatic cancer<br>Prostate cancer<br>Pathways in cancer	OPTN (0.67) testis<br>RNF11 (0.56) testis<br>TSC1 (0.52) testis	Not found
C8orf76	DLBC ↑<br>THYM ↑	CTDSP1, DISC1, HSPA8, KLC2, NOTCH3, SKP2, SLK, VRK1	MicroRNAs in cancer<br>Renal cell carcinoma<br>Breast cancer<br>Non-small cell lung cancer	HSPA8 (0.69) DLBC<br>KLC2 (0.57) DLBC<br>HSPA8 (0.59) thymoma	Not found
C9orf40	DLBC ↑<br>THYM ↑	CALM1, CDK2, HSPA1A, KPNA4, MOV10, SHMT2, **XPO1**	Proteoglycans in cancer<br>Pathways in cancer<br>MicroRNAs in cancer	CDK2 (0.69) thymoma<br>CDK2 (0.57) DLBC<br>XPO1 (0.58) DLBC	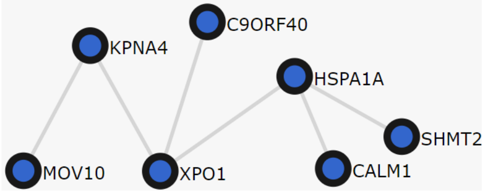
C9orf43	TGCT ↓	APP, DDB2, EZH2, PARP1	Transcriptional misregulation in cancer<br>Pathways in cancer<br>Viral carcinogenesis<br>MicroRNAs in cancer<br>Human T-cell leukemia virus 1 infection	EZH2 (0.59) testis	Not found
C9orf64	THYM ↑	BCAR1, ELAVL1, **FH**, LRRK2, **MAP3K1**, **MYC**, PKM, PRKD2, SLC16A2, TRIM28	Pathways in cancer<br>Kaposi sarcoma-associated herpesvirus infection<br>MicroRNAs in cancer	ELAVL1 (0.65) thymoma<br>LRRK2 (0.54) thymoma<br>MAP3K1 (0.72) thymoma	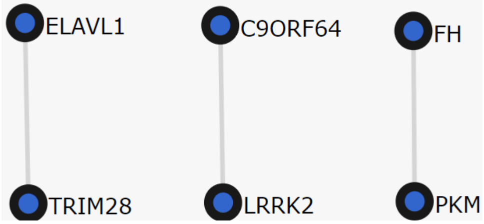

CXorf56	DLBC ↑<br>THYM ↑	APP, **BRD4**, CDC20, ELAVL1, **EZH2,** HAT1, **JUN**, **LMNA**, **MYC**, **NSD2**, OTUB1, **RECQL4**, **RET**. RPRD1B, SNW1, XRCC6	Transcriptional misregulation in cancer<br>Pathways in cancer<br>Hepatocellular carcinoma<br>Gastric cancer<br>Breast cancer<br>Breast cancer	APP (0.62) DLBC<br>BRD4 (0.63) DLBC<br>ELAVL1 (0.73) DLBC<br>EZH2 (0.62) DLBC<br>HAT1 (0.72) DLBC<br>NSD2 (0.71) DLBC<br>RPRD1B (0.59) DLBC<br>SNW1 (0.61) DLBC<br>RPRD1B (0.87) thymoma<br>HAT1 (0.66) THYM<br>ELAVL1 (0.6) THYM<br>BRD4 (0.77) THYM<br>APP (0.63) THYM	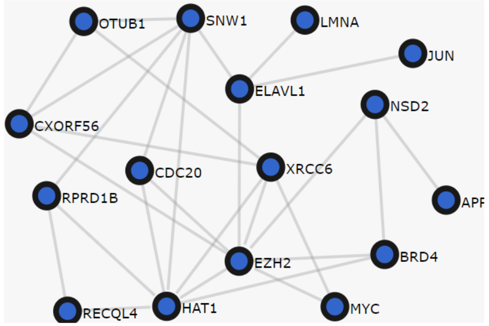
C10orf67	TGCT ↓	**BCL2L1,** BCL2L2, NOD2, TNF	Pathways in cancer<br>Small cell lung cancer<br>Pancreatic cancer	BCL2L1 (0.63) testis	
C11orf49	DLBC ↑<br>THYM ↑	**CCNE1,** EIF3F, **FH,** MIB1, NDN, PHF1, **PICALM,** PRKACB, **PRKAR1A,** PSMA1, UBE3A, UBQLN4	Pathways in cancer<br>Prostate cancer	NDN (0.61) thymoma<br>EIF3F (0.57) thymoma	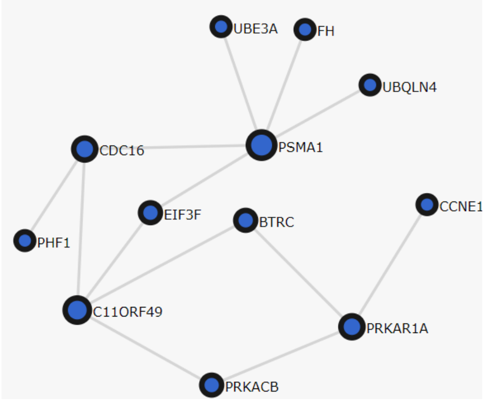
C11orf52	OV ↑<br>SKCM ↓<br>UCES ↑	ESR2, **HRAS**, APP	Pathways in cancer<br>Breast cancer	Not found	Not found

C11orf65	TGCT ↓	AHCYL1, CALM2, CALM3, **CDH1**, CDK5R1, DAPK1, DLST, SUMO1, UBE2T	Pathways in cancer<br>Proteoglycans in cancer<br>Glioma<br>Renal cell carcinoma	CALM3 (0.81) testis<br>CDK5R1 (0.73) testis	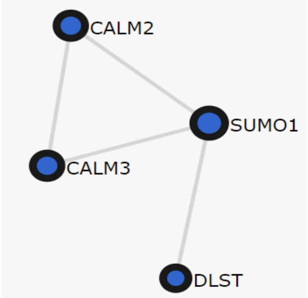
C11orf98	DLBC ↑<br>PAAD ↑<br>THYM ↑	ESR2, **FOXA1,** GSK3B, **JUN,** ESR1, GSK3A, **MYC,** WWP2	Pathways in cancer<br>Breast cancer<br>Hepatocellular carcinoma<br>Prostate cancer<br>Colorectal cancer	Not found	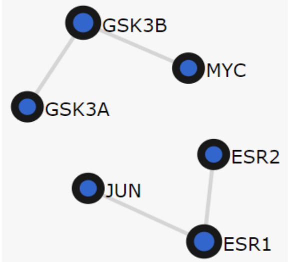
C12orf29	DLBC ↑<br>THYM ↑	BCAR1, LRRK2, MAPK12, RNF123	Pathways in cancer<br>Proteoglycans in cancer<br>Viral carcinogenesis	LRRK2 (0.58) DLBC<br>LRRK2 (0.50) thymoma	Not found
C12orf4	THYM ↑	CASP6, GAPDHS, HMOX2, LAMP2, **SRC**, SH3GLB1, TBCK	Pathways in cancer<br>Proteoglycans in cancer<br>Renal cell carcinoma<br>Pancreatic cancer<br>Non-small cell lung cancer<br>PD-L1 expression and PD-1 checkpoint pathway in cancer<br>Central carbon metabolism in cancer	LAMP2 (0.66) thymoma<br>SRC (0.51) thymoma<br>SH3GLB1 (0.83) thymoma<br>TBCK (0.84) thymoma	Not found

C12orf45	DLBC ↑<br>THYM ↑	BCAR1, KPNA2, RUVBL1, SMARCAD1, BMX, KPNA1, PRKAA1, RUVBL2, SARS1, SPAG9, TRIM25, WASHC2C	Viral carcinogenesis<br>Transcriptional misregulation in cancer	Not found	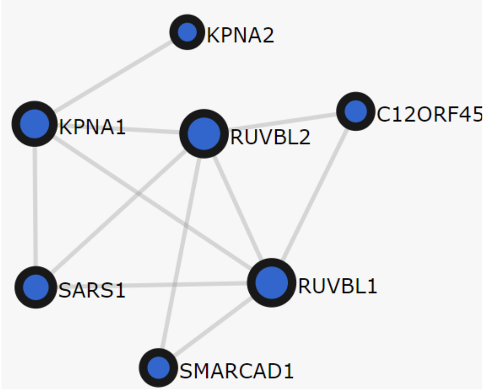
C12orf49	DLBC ↑<br>PAAD ↑	ATP13A1, ATP2B2, CLTCL1, EIF2B5, ELAVL1, FAM8A1, FBXO2, MBTPS1, MTCH2, SCAP, SKP1, SREBF1, SREBF2, SYVN1, TP53	Prostate cancer<br>Human T-cell leukemia virus 1 infection<br>MicroRNAs in cancer<br>Pancreatic cancer<br>Kaposi sarcoma-associated herpesvirus infection	ELAVL1 (0.59) DLBC<br>FAM8A1 (0.53) DLBC MBTPS1 (0.55) DLBC<br>SKP1 (0.56) DLBC<br>SREBF2 (0.52) DLBC	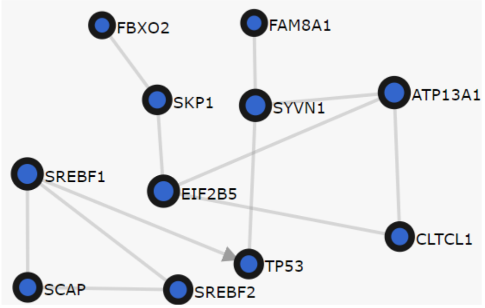
C15orf39	TGCT ↓	APP, CARD8, CDC16, CDH19, CTBP1, CTBP2, EGLN3, HSPA1A MAD2L1, NCAPH2, **ZEB1**, ZEB2, **XPO1**	Human T-cell leukemia virus 1 infection<br>MicroRNAs in cancer<br>Hepatocellular carcinoma<br>Pathways in cancer<br>Gastric cancer<br>Chronic myeloid leukemia	ZEB1 (0.62) testis<br>XPO1 (0.53) TGCT	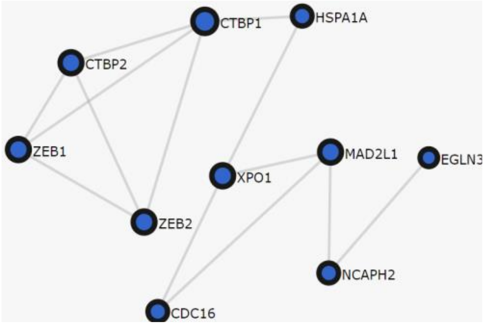
C16orf54	LAML ↑	DIP2A, LPAR3, PIK3R3, EFNB1	Non-small cell lung cancer<br>Breast cancer<br>MicroRNAs in cancer<br>Prostate cancer	Not found	Not found

			Gastric cancer<br>Endometrial cancer		
C16orf71	TGCT ↓	AURKA, CHFR, **CREB1**, **EZH2**, MEIS2, ARRB2, **CDKN2C**, DUSP3, MEIS1, NAMPT, **PBX1**, PLEKHG4, **PTEN**, RNF138, SEH1L, STRADA, TYK2	Transcriptional misregulation in cancer<br>Pathways in cancer<br>Prostate cancer<br>Pancreatic cancer<br>Chronic myeloid leukemia<br>Breast cancer	AURKA (0.81) testis<br>CHFR (0.57) testis<br>CREB1 (0.72) testis<br>EZH2 (0.66) testis<br>MEIS2 (0.52) TGCT<br>MEIS1 (0.55) TGCT<br>RNF138 (0.67) testis<br>STRADA (0.83) testis	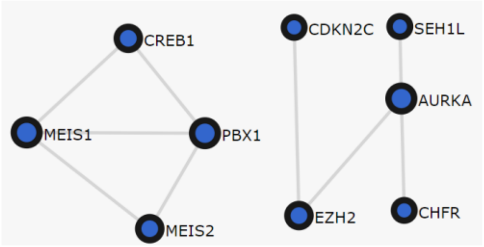
C16orf87	DLBC ↑<br>THYM ↑	HDAC1, CDC16, ANAPC10, ANAPC16, ELAVL1, ANAPC5, FBXO5, ANAPC7, YWHAG, ANAPC1, CDC26, CDC27, H2AX, ANAPC2, CDC20, ANAPC13, NEK2, HDAC2, MOV10, FZR1	Pathways in cancer<br>Viral carcinogenesis<br>Small cell lung cancer<br>Breast cancer<br>Gastric cancer<br>Prostate cancer<br>Acute myeloid leukemia	HDAC1 (0.62) thymoma<br>CDC16 (0.54) DLBC<br>ANAPC10 (0.65) DLBC; ANAPC10 (0.87) thymoma<br>ANAPC16 (0.54) thymoma<br>ELAVL1 (0.61) DLBC<br>ELAVL1 (0.63) thymoma<br>FBXO5 (0.57) DLBC<br>YWHAG (0.61) DLBC<br>YWHAG (0.54) thymoma<br>ANAPC1 (0.65) DLBC<br>ANAPC1 (0.54) thymoma<br>CDC27 (0.71) DLBC<br>CDC27 (0.70) thymoma<br>ANAPC13 (0.70) thymoma<br>NEK2 (0.56) DLBC<br>HDAC2 (0.59) DLBC<br>FZR1 (0.59) DLBC<br>FZR1 (0.67) thymoma	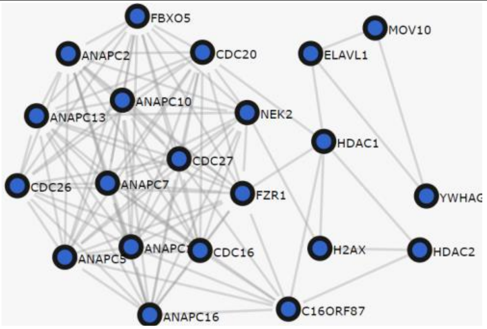

C16orf90	TGCT ↓	BIRC2, NDFIP1, RNF123, XIAP, NDFIP2, TRAF2	Small cell lung cancer<br>Pathways in cancer<br>PD-L1 expression and PD-1 checkpoint pathway in cancer<br>Prostate cancer<br>Pancreatic cancer<br>Colorectal cancer	RNF123 (0.55) testis<br>NDFIP2 (0.65) testis	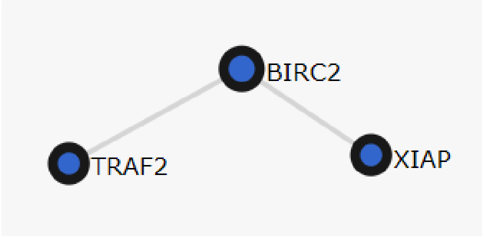
C17orf47	TGCT ↓	ADRM1, APP, AREL1, **BCL2,** CCNA1, DYNLL2, FMR1, HCK, NUP214, PARP1, **PPM1D,** SIAH1, **TSC2**, WWP2	Pathways in cancer<br>Gastric cancer<br>MicroRNAs in cancer<br>Breast cancer<br>Colorectal cancer<br>Hepatocellular carcinoma<br>Endometrial cancer	ADRM1 (0.65) testis<br>CCNA1 (0.8) testis<br>NUP214 (0.8) testis<br>PPM1D (0.83) testis<br>SIAH1 (0.71) testis	Not found
C19orf18	TGCT ↓	ACVR2A, APP, **BMPR1A,** **FGFR1,** **FGFR2,** NDFIP1, TNFRSF10B	Pathways in cancer<br>Gastric cancer<br>Proteoglycans in cancer<br>Breast cancer<br>Colorectal cancer<br>Hepatocellular carcinoma<br>Basal cell carcinoma	Not found	Not found
C19orf44	TGCT ↓<br>THYM ↑	BIRC2, DYNLL1, GAPDHS, PTK2B, TRIM25, XPO1	PD-L1 expression and PD-1 checkpoint pathway in cancer<br>Proteoglycans in cancer<br>Kaposi sarcoma-associated herpesvirus infection	GAPDHS (0.83) testis<br>TRIM25 (0.56) testis	Not found

C19orf53	DLBC ↑<br>THYM ↑	APEX1, APP, CTTN, **ESR1,** GRB10, GRB7, HNRNPU, KDM1A, POU5F1, **SDHA**	Pathways in cancer<br>Breast cancer<br>Prostate cancer<br>Gastric cancer<br>Transcriptional misregulation in cancer<br>Hepatocellular carcinoma	<u>SDHA (0.82) thymoma</u>	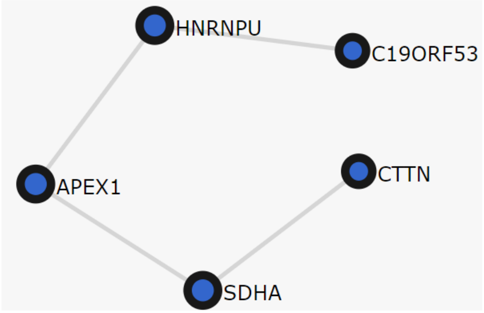
C20orf27	DLBC ↑	ARHGEF7, CHEK2, CSK, EIF2AK4, FERMT2, LMTK2, PPP1CA, PPP1CB, PPP1CC, PPP1R3A, PPP1R3B, PPP1R9A, PPP1R9B, RRM2, SCRIB, TERF2, ZFYVE9	Proteoglycans in cancer<br>Pathways in cancer<br>Gastric cancer<br>Breast cancer<br>Glioma<br>Hepatocellular carcinoma<br>Endometrial cancer	Not found	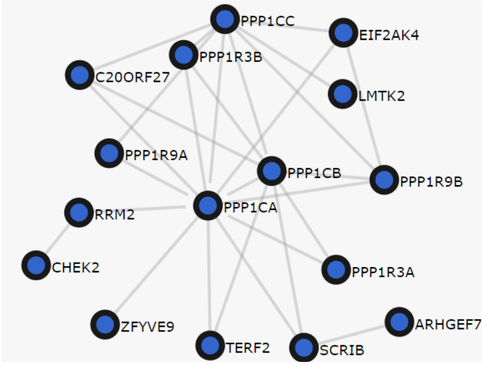

Notes: protein partners encoded by cancer driver genes are highlighted with bold; correlation coefficient values > 0.8 are underlined.
* PathwAX II server – pathway class: diseases; ** GEPIA2 server; *** FunCoup v.5.0 server;
# (↑ and ↓), up- and down-regulation of gene expression

Table S4. Over-representation analysis of ORF-proteins subinteractomes

ORF-protein	Interaction partners	CH-score*	Functional terms**
C1orf21	27	4.1	WP3932~focal adhesion-PI3K-Akt-mTOR-signaling pathway (26%)<br>GO:0016311~dephosphorylation (22%)<br>GO:1903293~phosphatase complex (22%)<br>GO:0019208~phosphatase regulator activity (19%)
C1orf115	28	3.2	GO:0016741~transferase activity, transferring one-carbon groups (21%)<br>GO:0008213~protein alkylation (21%)<br>GO:0034708~methyltransferase complex (14%)<br>GO:0032182~ubiquitin-like protein binding (14%)<br>GO:0005200~structural constituent of cytoskeleton (14%)<br>GO:0019787~ubiquitin-like protein transferase activity (14%)<br>GO:0018195~peptidyl-arginine modification (14%)
C1orf123	40	2.8	Not found
C1orf131	43	4.9	GO:0022613~ribonucleoprotein complex biogenesis (25%)
C1orf198	75	2.4	GO:0051493~regulation of cytoskeleton organization (15%)<br>GO:0097193~intrinsic apoptotic signaling pathway (10%)
C1orf210	41	2.7	GO:0016197~endosomal transport (15%)<br>GO:0038061~NIK/NF-kappaB signaling (10%)<br>WP366~TGF-beta Signaling Pathway (< 10%)
C1orf226	48	3.3	PA443653~cell transformation, neoplastic (17%)<br>GO:0016607~nuclear speck (17%)<br>GO:0045930~negative regulation of mitotic cell cycle (15%)<br>GO:1901987~regulation of cell cycle phase transition (15%)<br>PA443358~aneuploidy (15%)<br>GO:0048511~rhythmic process (10%)<br>GO:0008013~beta-catenin binding (10%)
C2orf16	13	5.4	GO:0030496~midbody (23%)<br>WP61~Notch Signaling Pathway Netpath (23%)
C2orf74	32	2.8	GO:0016311~dephosphorylation (31%)<br>GO:0019902~phosphatase binding (28%)<br>GO:1903293~phosphatase complex (19%)
C2orf88	19	5.2	GO:0051018~protein kinase A binding (37%)<br>GO:0044441~ciliary part (37%)<br>GO:0030104~water homeostasis (26%)<br>GO:0051348~negative regulation of transferase activity (21%)<br>WP4249~Hedgehog signaling pathway (16%)<br>PA446628~adrenocortical adenoma (16%)
C3orf62	22	2.7	GO:1901987~regulation of cell cycle phase transition (23%)<br>GO:0045787~positive regulation of cell cycle (18%)<br>GO:0007568~aging (18%)<br>GO:0033613~activating transcription factor binding (18%)<br>WP366~TGF-beta signaling pathway (18%)
C4orf17	18	4.2	GO:0016569~covalent chromatin modification (33%)
C4orf19	21	2.7	GO:0031098~stress-activated protein kinase signaling cascade (26%)<br>GO:0050839~cell adhesion molecule binding (26%)
C4orf46	26	3.8	GO:0098793~presynapse (19%)<br>PA446061~warts (19%)<br>GO:0005819~spindle (15%)<br>GO:0035329~hippo signaling (12%)
C5orf24	48	4.0	PA444750~leukemia (19%)

			GO:0001228~DNA-binding transcription activator activity, RNA polymerase II-specific (19%)
C6orf132	7	5.7	Not found
C6orf222	38	6.3	GO:1901987~regulation of cell cycle phase transition (21%)<br>GO:0010498~proteasomal protein catabolic process (18%)<br>GO:0000151~ubiquitin ligase complex (16%)<br>WP179~cell cycle (16%)<br>PA443937~drug toxicity (16%)<br>PA443358~aneuploidy (16%)<br>GO:0090559~regulation of membrane permeability (14%)<br>CORUM complex ID 93~anaphase-promoting complex (11%)
C8orf48	23	5.2	GO:1902532~negative regulation of intracellular signal transduction (26%)<br>HP:0010786~urinary tract neoplasm (17%)
C8orf76	24	3.2	GO:0050839~cell adhesion molecule binding (25%)
C9orf40	27	2.6	Not found
C9orf43	10	4.0	Not found
C9orf64	36	2.8	Not found
CXorf56	61	2.6	GO:0005681~spliceosomal complex (30%)<br>CORUM complex ID 351~spliceosome (28%)<br>GO:0008380~RNA splicing (21%)<br>GO:1901987~regulation of cell cycle phase transition (11%)<br>C0029463~osteosarcoma (10%)<br>GO:0004386~helicase activity (< 10%)
C10orf67	13	3.1	WP254~apoptosis (23%)
C11orf49	56	2.7	GO:0005874 ~microtubule (18%)<br>GO:0044782~cilium organization (16%)<br>GO:1901987~regulation of cell cycle phase transition (13%)<br>GO:0015631~tubulin binding (13%)<br>GO:0016197~endosomal transport (11%)
C11orf52	9	3.3	Not found
C11orf65	27	3.3	GO:0010038~response to metal ion (19%)
C11orf98	17	4.7	GO:0001085~RNA polymerase II transcription factor binding (29%)<br>GO:0051090~regulation of DNA-binding transcription factor activity (29%)<br>WP4262~breast cancer pathway (29%)<br>GO:0002064~epithelial cell development (24%)<br>GO:0043254~regulation of protein complex assembly (24%)
C12orf29	8	5.0	Not found
C12orf4	16	4.4	Not found
C12orf45	28	4.3	GO:0006310~DNA recombination (18%)
C12orf49	49	3.0	GO:0034976~response to endoplasmic reticulum stress (16%)<br>GO:0008202~steroid metabolic process (14%)<br>GO:0031227~intrinsic component of endoplasmic reticulum membrane (12%)<br>GO:0000041~transition metal ion transport (11%)<br>GO:0044322~endoplasmic reticulum quality control compartment (11%)<br>GO:0051082~unfolded protein binding (11%)<br>CORUM complex ID 6859~HRD1 complex (11%)
C15orf39	65	2.0	GO:0048285~organelle fission (12%)<br>CORUM complex ID 642~CtBP complex (12%)<br>PA445644~shock (12%)
C16orf54	16	2.5	Not found
C16orf71	42	4.0	PA444761~leukemia, myeloid (17%)<br>GO:0031647~regulation of protein stability (15%)<br>GO:0007517~muscle organ development (15%)

C16orf87	65	3.1	GO:1901987~regulation of cell cycle phase transition (26%)<br>GO:0000151~ubiquitin ligase complex (23%)<br>WP179~cell cycle (23%)<br>PA443358~aneuploidy (14%)<br>CORUM complex ID 96~anaphase-promoting complex (14%)<br>GO:0044815~DNA packaging complex (12%)<br>GO:0016458~gene silencing (< 10%)<br>GO:0051321~meiotic cell cycle (< 10%)<br>GO:0070491~repressing transcription factor binding (< 10%)<br>GO:0019902~phosphatase binding (< 10%)
C16orf90	9	6.7	GO:1903320~regulation of protein modification by small protein conjugation or removal (44%)<br>GO:0019787~ubiquitin-like protein transferase activity (44%)<br>WP254~apoptosis (33%)
C17orf47	45	3.1	GO:0098793~presynapse (18%)<br>GO:0044772~mitotic cell cycle phase transition (16%)<br>GO:0044389~ubiquitin-like protein ligase binding (13%)<br>GO:0031072~heat shock protein binding (13%)<br>GO:0006979~response to oxidative stress (11%)
C19orf18	15	4.7	GO:0043235~receptor complex (33%)<br>GO:0007498~mesoderm development (27%)<br>GO:0019199~transmembrane receptor protein kinase activity (27%)
C19orf44	27	2.2	Not found
C19orf53	21	4.8	GO:0010608~posttranscriptional regulation of gene expression (38%)<br>GO:0035770~ribonucleoprotein granule (23%)<br>GO:0016607~nuclear speck (23%)<br>GO:0003729~mRNA binding (23%)<br>GO:0040029~regulation of gene expression, epigenetic (19%)
C20orf27	65	2.6	GO:0016311~dephosphorylation (22%)<br>GO:0019208~phosphatase regulator activity (12%)<br>GO:1903293~phosphatase complex (11%)<br>GO:0005976~polysaccharide metabolic process (11%)

*CH-score means a frequency of occurrence of cancer hallmarks associated proteins per any ten proteins in each subinteractome of an ORF-protein

**functional term covering > 10% of a total number of protein partners is considered as significant (FDR < 0.1)

C10orf120	*C15orf61*	*C19orf67*	*C2orf42*	*C6orf163*
C10orf53	*C15orf65*	*C19orf84*	*C2orf70*	*C6orf201*
C10orf55	*C16orf46*	*C1orf105*	*C2orf73*	*C6orf99*
C10orf62	*C16orf71*	*C1orf141*	*C2orf78*	*C7orf31*
C10orf67	*C16orf78*	*C1orf146*	*C3orf20*	*C7orf61*
C10orf88	*C16orf82*	*C1orf158*	*C3orf22*	*C7orf66*
C11orf16	*C16orf86*	*C1orf159*	*C3orf30*	*C7orf71*
C11orf42	*C16orf90*	*C1orf185*	*C3orf62*	*C8orf48*
C11orf53	*C16orf92*	*C1orf189*	*C3orf84*	*C8orf74*
C11orf65	*C16orf96*	*C1orf94*	*C4orf17*	*C8orf89*
C11orf71	*C16orf97*	*C20orf141*	*C4orf36*	*C9orf131*
C11orf88	*C17orf112*	*C20orf144*	*C4orf45*	*C9orf153*
C11orf91	*C17orf47*	*C20orf173*	*C4orf47*	*C9orf43*
C11orf94	*C17orf50*	*C20orf187*	*C4orf51*	*C9orf50*
C11orf97	*C17orf64*	*C20orf78*	*C5orf47*	*C9orf57*
C12orf42	*C17orf98*	*C22orf15*	*C5orf49*	*CXorf49*
C12orf50	*C18orf63*	*C22orf23*	*C5orf52*	*CXorf51B*
C12orf60	*C19orf18*	*C22orf31*	*C5orf58*	*CXorf58*
C12orf71	*C19orf47*	*C22orf42*	*C5orf60*	*CXorf65*
C15orf39	*C19orf57*	*C2orf16*	*C6orf118*	*CXorf66*

Figure S1. Testicular Germ Cell Tumors (TGCT)-specific 100-gene expression signature.

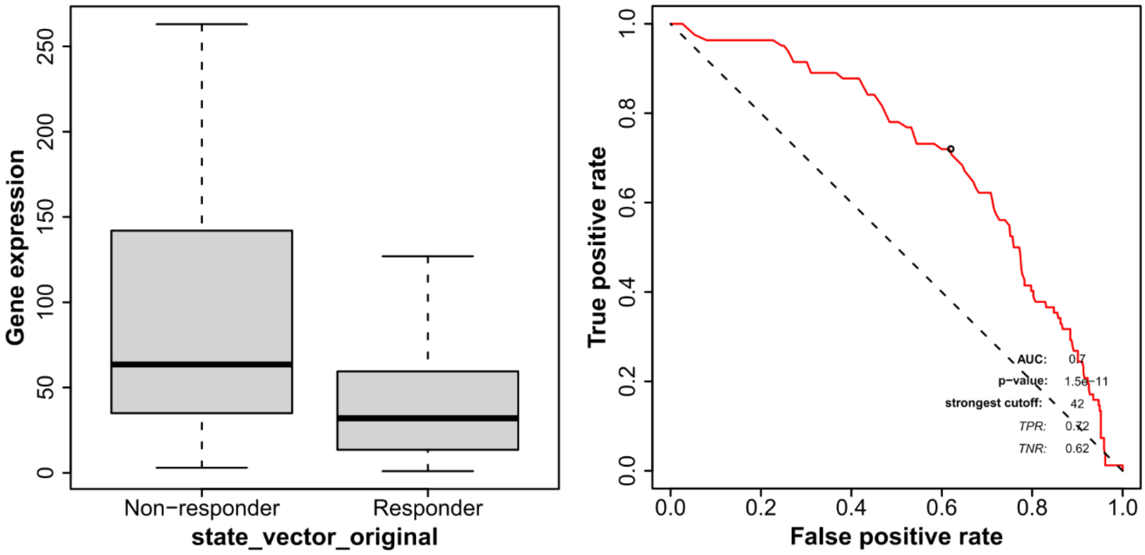

Figure S2. Predictive significance of *C22orf42* gene expression levels: responses were based on relapse free survival at 6 months period; median values of gene expression levels are 32 and 64 for 415 responders and 82 non-responders, respectively. The figure shows the original data output returned from the ROC-plotter server.

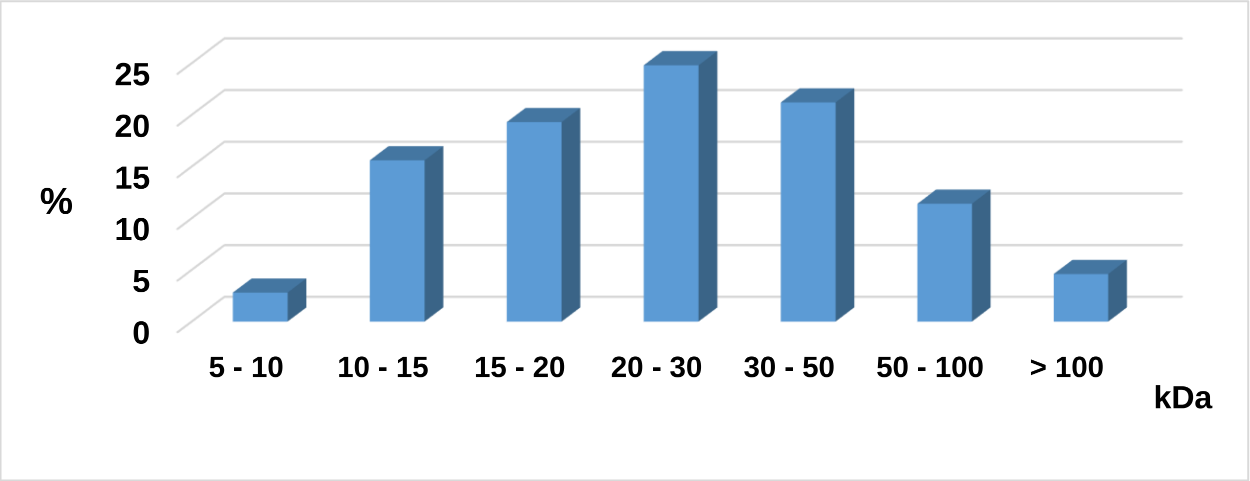

Figure S3. The distribution of ORF-proteins according to their molecular weight.

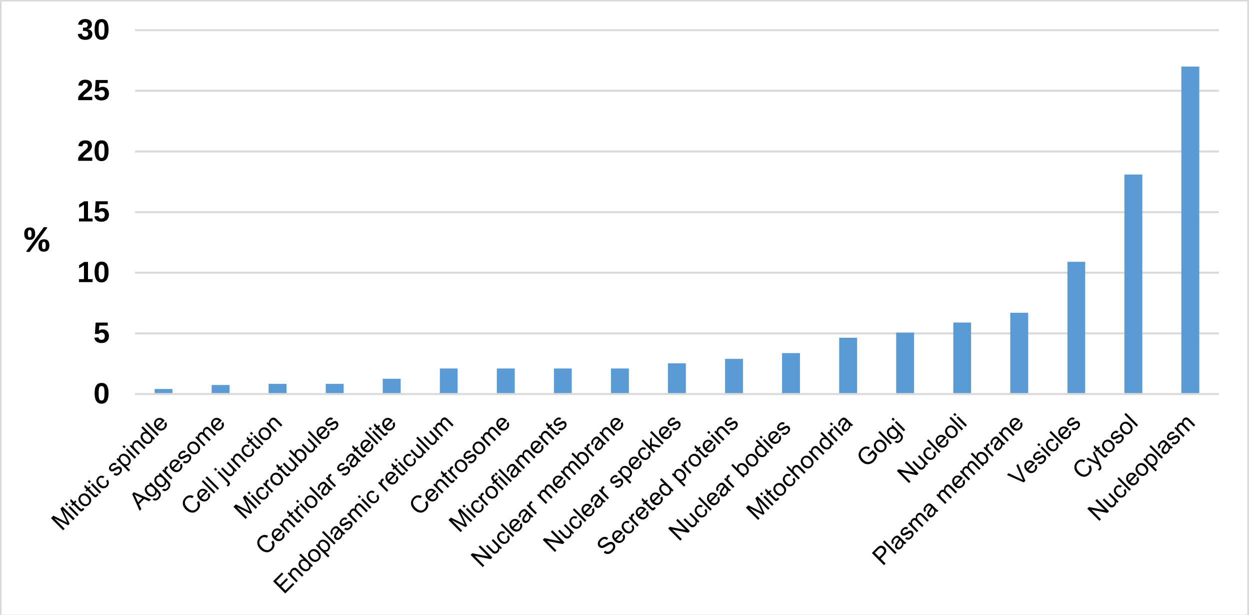

Figure S4. The distribution of ORF-proteins according to their subcellular localization.
